# An Image-Based Computational Fluid Dynamics Study of Mitral Regurgitation in Presence of Prolapse

**DOI:** 10.1007/s13239-023-00665-3

**Published:** 2023-04-17

**Authors:** Lorenzo Bennati, Christian Vergara, Vincenzo Giambruno, Ivan Fumagalli, Antonio Francesco Corno, Alfio Quarteroni, Giovanni Puppini, Giovanni Battista Luciani

**Affiliations:** 1 Department of Surgery, Dentistry, Pediatrics, and Obstetrics/Gynecology, University of Verona, Piazzale Ludovico Antonio Scuro 10, 37134 Verona, Italy; 2 LaBS, Dipartimento di Chimica, Materiali e Ingegneria Chimica “Giulio Natta”, Politecnico di Milano, Piazza Leonardo da Vinci 32, 20133 Milan, Italy; 3 Division of Cardiac Surgery, Department of Surgery, Dentistry, Pediatrics, and Obstetrics/Gynecology, University of Verona, O. C. M. Piazzale Stefani 1, 37126 Verona, Italy; 4 MOX, Dipartimento di Matematica, Politecnico di Milano, Piazza Leonardo da Vinci 32, 20133 Milan, Italy; 5 Children’s Heart Institute, McGovern Medical School, UT Health, 6431 Fannin Street, Houston, TX 77030 USA; 6 École Polytechnique Fédérale de Lausanne, Rte Cantonale, 1015 Lausanne, Switzerland; 7 Department of Radiology, University of Verona, O. C. M. Piazzale Stefani 1, 37126 Verona, Italy

**Keywords:** Mitral valve regurgitation, Image-based computational fluid dynamics, Wall shear stresses, Cine-MRI images

## Abstract

**Purpose:**

In this work we performed an imaged-based computational study of the systolic fluid dynamics in presence of mitral valve regurgitation (MVR). In particular, we compared healthy and different regurgitant scenarios with the aim of quantifying different hemodynamic quantities.

**Methods:**

We performed computational fluid dynamic (CFD) simulations in the left ventricle, left atrium and aortic root, with a resistive immersed method, a turbulence model, and with imposed systolic wall motion reconstructed from Cine-MRI images, which allowed us to segment also the mitral valve. For the regurgitant scenarios we considered an increase of the heart rate and a dilation of the left ventricle.

**Results:**

Our results highlighted that MVR gave rise to *regurgitant jets* through the mitral orifice impinging against the atrial walls and scratching against the mitral valve leading to high values of wall shear stresses (WSSs) with respect to the healthy case.

**Conclusion:**

CFD with prescribed wall motion and immersed mitral valve revealed to be an effective tool to quantitatively describe hemodynamics in case of MVR and to compare different regurgitant scenarios. Our findings highlighted in particular the presence of transition to turbulence in the atrium and allowed us to quantify some important cardiac indices such as cardiac output and WSS.

## Introduction

Mitral valve regurgitation (MVR) is a congenital or acquired pathological condition of the mitral valve leading to an incomplete closure of the leaflets during the systolic phase, causing a retrograde blood flow from the left ventricle to the left atrium. The major complications are atrial fibrillation or flutter, pulmonary arterial hypertension and heart failure [69]. Epidemiological studies estimated that MVR occurs in up to 10% of the worldwide population [41].

The quantitative assessment of blood dynamics quantities, such as pressure, velocity and stresses, in presence of MVR during the systolic phase is crucial for the understanding of the phenomena and to elaborate the diagnosis. For example, the regurgitant volume (RV) and the regurgitant fraction (RF) can help physicians in assessing the severity of the pathological condition [73] and potentially helping the design of treatments [33, 54].

Computational methods in realistic cardiac geometries represent a non invasive way to provide blood dynamics information [26] and help the surgeons, e.g., by suggesting possible clinical indications for the surgical treatment of the systolic anterior motion [29] and of septal myectomy for hypertrophic obstructive cardiomyopathy [84].

Computational studies investigating the mitral valve and its interaction with the blood flow have been proposed since the early 2000s, and can be divided into two categories: (i) fluid–structure interaction (FSI) models and (ii) computational fluid dynamics (CFD) models with imposed wall motion.

Regarding the FSI approach, some authors investigated the mitral valve dynamics in physiological conditions [76, 3, 44, 40, 28, 48, 77], in case of calcific leaflets [40], to model surgical interventions as the neochordae replacement [27], and to account for MVR [35, 74]. In Ref. [74], MVR has been modeled by means of an idealized diode whilst in Ref. [35] the healthy configuration has been deformed. The majority of these works [76, 3, 44, 40, 28, 48] used the immersed boundary method (IBM), proposed in Refs. [48, 5].

In order to reduce the high computational costs of FSI simulations, CFD models with imposed ventricular and leaflets motion provided by dynamic imaging have also been considered. Such models require a great effort in elaborating the dynamic images and in merging them with CFD. However, they noticeably simplify the problem from the modeling point of view.

The majority of such works are based on images provided by ultrasound (US) techniques (like 3D echocardiography). 3D Echo has the advantage of providing high temporal resolution, particularly useful when resolving the rapid movements of the mitral valve leaflets [84]. For example, in Refs. [55, 47] the authors prescribed both the ventricle and the mitral valve motion to study the ventricular flow in physiological conditions. In Ref. [53], the authors reconstructed the ventricle size and shape from computer tomography (CT) scans and the mitral valve from US to predict the outcomes of the mitraclip, a surgical technique used to restore the mitral valve function.

Another dynamic imaging technique used for ventricular CFD is Cine-MRI. However, such technique has been used to reconstruct only the motion of the ventricle whereas different models of the mitral valve have been considered. For example, we cite Ref. [18] where the authors investigated the effect of incorporating an idealized mitral valve in patients affected by pulmonary artery hypertension, and Refs. [29, 88] where a template of mitral valve was included to study the systolic anterior motion and the hypertrophic cardiomyopathy.

Regarding the study of the MVR by CFD with imposed motion, we cite Refs. [10, 16], where the authors tested and compared different types of mitral valve prolapse from US images and different degrees of MVR from CT images.

This work aims at providing an original study of the systolic blood dynamics in the left ventricle in physiological and MVR conditions. For this purpose, we introduced some contributions in the context of computational models for ventricular fluid-dynamics. First, we adapted to the systolic phase (closed mitral valve) a method based on radial sampling Cine-MRI acquisitions developed in Ref. [67] for the diastolic phase and applied to a structural analysis. Second, we applied, for the first time, such reconstruction, from radial sampling Cine-MRI, to the context of a systolic CFD with ventricular motion, obtained by a (not radial) Cine-MRI acquisition. The third contribution of the paper consisted in the comparison of the systolic blood dynamics in physiological and MVR conditions, where for the latter case we considered two scenarios, i.e. an increment of the heart rate and a ventricular dilation [60].

## Materials and Methods

### Description of the Scenarios

In this work we provided a comparison of blood flow dynamics in scenarios with healthy and regurgitant mitral valves during the systolic phase.^1^ To do this, we considered the same geometric and moving conditions for the left ventricle, for the aorta, and for the left atrium. In this respect, we created three different virtual scenarios:

- *healthy* (H): we inserted a structurally normal mitral valve (taken from dedicated MRI images) and we considered a heart rate equal to 75 bpm/min^2^ (duration of the systolic phase $T_{\text {S}}=0.32$ s);
- *regurgitant with increased heart rate* (R1): we inserted a regurgitant mitral valve with a posterior leaflet prolapse of the P2 segment (reconstructed from dedicated MRI images) and we increased the basal heart rate to 90 bpm/min ($T_{\text {S}}=0.26$ s [32, 62]);
- *regurgitant with dilatation* (R2): we inserted the same regurgitant mitral valve of R1, with heart rate equal to 75 bpm/min, and we dilated all the geometry (ventricle, atrium, aorta).

The regurgitant scenarios represent different stages of the pathological evolution of the heart function in case of MVR due to prolapse, leading to a decrease of the cardiac output (CO). To compensate, in an *early stage* the heart rate spontaneously increases (R1) [60]. However, this leads to a reduction of the duration of the diastolic phase, making the coronary arteries perfusion less effective over the time. Thus, in a *medium-long term*, the early compensatory changes are gradually replaced by a remodeling process with dilation of the left ventricle and restoring of the basal heart rate (R2). The resultant increase of the stroke volume (SV) tends to compensate for the low CO [60].

### Acquisition of Cine-MRI Images

Cardiac Cine-MRI data were provided by the Department of Radiology of Borgo Trento Hospital, Verona, Italy. Ethical Review Board approval and informed consent were obtained from all patients.

The acquisitions were performed with the Achieva 1.5T (TX)-DS (Philips, Amsterdam, Netherlands) technology with the following characteristics:

- *Left ventricle*: (i) volumetric short-axis series made of 15 slices with thickness and distancing of 8 mm along the left ventricle main axis, with a spatial resolution of 1 mm; (ii) a set of single-slices, two-dimensional long-axis acquisitions on the so-called two-chamber and four-chamber planes, with space resolution of 1 mm and slice thickness of 8 mm. Both series had a time resolution equal to 30 frames/cardiac cycle. This protocol was carried out for a subject who did not present any alteration of the left ventricle wall and movement;
- *Mitral valves*: two-dimensional long-axis series of 18 evenly rotated (one every $10^{\circ }$) planes around the axis passing through the annular center and aligned with the left ventricle. Time resolution equals to 30 frames/cardiac cycle, spatial resolution equals to 1.25 mm, and slice thickness to 8 mm. This protocol was repeated for both a healthy patient and one with a regurgitant mitral valve.

In what follows we describe how such images were elaborated and reconstructed.

### Geometric Reconstruction of the Left Ventricular Endocardium

In this section we described the strategy followed to reconstruct the geometry and the displacement field of the left ventricular endocardium.

The starting point were the volumetric short-axis images, which are *ad hoc* series for the reconstruction of the left ventricular anatomy. However, one of the main disadvantages often characterizing such images in the daily clinical practice is the low resolution among slices (in our case about 8 mm). As a consequence it is difficult to capture some important findings of the heart contraction, as the longitudinal shortening of the ventricle. To account for this, in this work we applied the short–long axis merging (SLAM) algorithm, proposed in Refs. [88, 31], which consists in enhancing the short-axis images by merging them with long-axis acquisitions (2 and 4 chambers views). For each available time instant, this procedure requires three main steps:

1. creation of a new artificial image with the same dimensions of the short-axis view and with a uniform space resolution (1 mm in our case) in all three Cartesian directions;
2. for each voxel V of this new artificial image, identify, for each slice of the original Cine-MRI image, the nearest voxel, and compute the distance $d^{\text {V}}$ between the two;
3. compute the weighted average of the values of the voxels identified at point 2, where the weights depend on $d^{\text {V}}$, and assign it as a gray level of V.

These steps lead to an enhanced time-dependent series of volumetric images with a uniform space resolution of 1 mm in all directions, including the cross-slice one.

Starting from these enhanced images, we segmented and reconstructed the shape of the endocardium and epicardium at six instants from the end diastolic (ED) to the end systolic (ES) configuration, by adopting the semi-manual segmentation algorithm proposed in Ref. [7] and implemented in the Medical Image Toolkit (MITK) open-source software (www.mitk.org) [11, 22]. This algorithm is based on a manual identification of the endocardial and epicardial 2D contours on each slice. Then, a smooth 3D surface is created by a 3D radial basis function interpolation of the 2D contours, based on the intensity of the image.

After, we performed a Boolean difference between the epicardium and the endocardium, in order to extract the region of the endocardium from the apex to the level of the valvular ring by using the Vascular Modeling Toolkit (VMTK) (www.vmtk.org) [46] and suitable tools for cardiac mesh generation [61]. We point out that during the identification of the ventricular endocardial contour, we included also the area occupied by the papillary muscles, in order to obtain a regular surface. This is a common choice adopted also in Refs. [29, 88, 78].

These steps were repeated for all the six frames, obtaining surface meshes made of triangles, representing the configurations of the endocardium during its contraction phase. Then, we registered the displacement needed to pass from a configuration to another one by exploiting the non-affine B-splines algorithm implemented in the Elastix open source library (http://elastix.isi.uu.nl), consisting in the minimization of the difference between two images under the assumption that they represent the same region, i.e. the same bounding box [86]. In particular, in our work, chosen a reference configuration, this was deformed to recover all the other frames. Calibration of registration parameters was performed in order to avoid possible formation of skewed triangles. The registration was run in serial on a Linux working station. This allowed us to compute the displacement fields (with respect to the ES configuration) at the six different time instants.

### Inclusion of Left Atrium, Aortic Valve, and Aorta

**Fig. 1:**
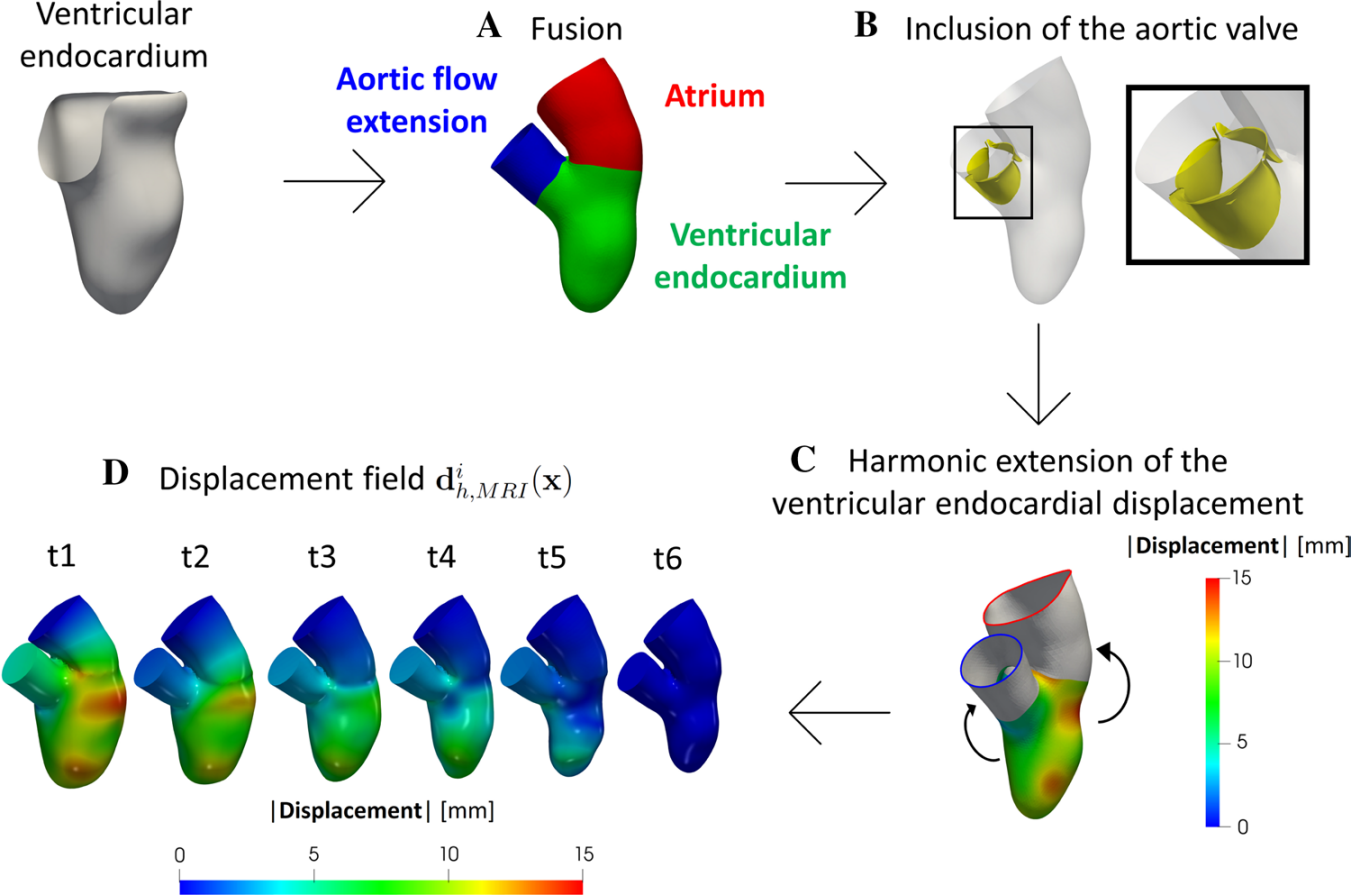
Steps to complete the geometry and its displacement. (A) Union of the atrium with the ventricular endocardium and addition of an aortic flow extension. (B) Inclusion of a template of the aortic valve (as an example, here we reported the open configuration). (C) Harmonic extension of the ventricular endocardial displacement for the atrium and for the aorta. (D) Geometries of the six current configurations together with the corresponding magnitude of the displacement field ${\mathbf {d}}_{{\text {h}},{\text {MRI}}}^{i}({\mathbf {x}})$ with respect to the ES instant

We completed the ventricular geometry by adding left atrium, aortic root and aortic valve. The strategy adopted is showed in Fig. 1.

Since the ventricular Cine-MRI images at disposal were not sufficient to allow a 3D reconstruction of the left atrium, aorta and aortic valve, that are only partially visible in long-axis images, we considered a template geometry of the internal atrial surface and the aortic valve from the *Zygote solid 3D heart model*, a complete geometry reconstructed from CT scans representing an average healthy heart (https://www.zygote.com). In particular, we merged such templates to the ES configuration of the reconstructed ventricular endocardium. For the healthy case H, we started our simulation at the aortic valve opening instant $t_{0}$ since we could not simulate the isovolumic phases. Indeed, the pressure, when both the valves are closed, is not uniquely defined due to the absence of a stress condition on a part of the fluid boundary domain (like, instead, happens at the atrial outflow in the regurgitant cases) [38, 17]. For the regurgitant cases, instead, general clinical observations show that MVR lowers the ventricular pressure until to be smaller than the aortic one, delaying the aortic valve opening. Thus, in R1 and R2, both valves are in their closed configuration at the initial time $t_{0}$ in order to prevent an inward flow from the aorta to the left ventricle at the beginning of systole. In this case, owing to the systolic stress condition at the mitral orifice due to the atrial pressure, we are able to start the simulation from the configuration where both valves are closed.

Moreover, we generated an aortic flow extension of the left ventricle outflow tract to account for the aorta (steps A and B in Fig. 1). These operations were done by using suitable algorithms in VMTK which, in particular, allowed us to connect harmonically two surfaces (*vmtksurfaceharmonicconnector*), to project a displacement field on a surface (*vmtksurfaceprojection*), and to solve a harmonic extension problem (*vmtksurfaceharmonicextension*) [61]. This allowed us to obtain a surface mesh of the complete geometry (ventricular endocardium, left atrium and aorta) at the ES configuration.

Then, to provide a displacement field also to the atrium and the aortic root, we harmonically extended the ventricular endocardial displacement to the corresponding surfaces (step C in Fig. 1). The reason why we selected an harmonic extension was due to the smooth solution provided by this operator, thus guaranteeing an homogeneous overall displacement. In such a way, a displacement field was defined for each point of the complete mesh at each of the six frames. After, we performed a L$^{2}$ projection of such data on the computational mesh used to perform Finite Elements simulations (see next section) to obtain the functions ${\mathbf {d}}_{{\text {h}}, {\text {MRI}}}^{i}({\mathbf {x}}),\,i=1,\ldots ,6$ [see step D in Fig. 1 where we reported the six configurations obtained together with the magnitude of ${\mathbf {d}}_{{\text {h}}, {\text {MRI}}}^{i}({\mathbf {x}})$]. Notice that the first instant (t1) refers to the ED configuration, whereas the last one (t6) to the ES configuration.

### Geometric Reconstruction of the Mitral Valve

In this section we reported the steps followed to reconstruct the two mitral valves at disposal in the systolic configuration and to build the three virtual scenarios under study (see “Description of the Scenarios” section).

**Fig. 2:**
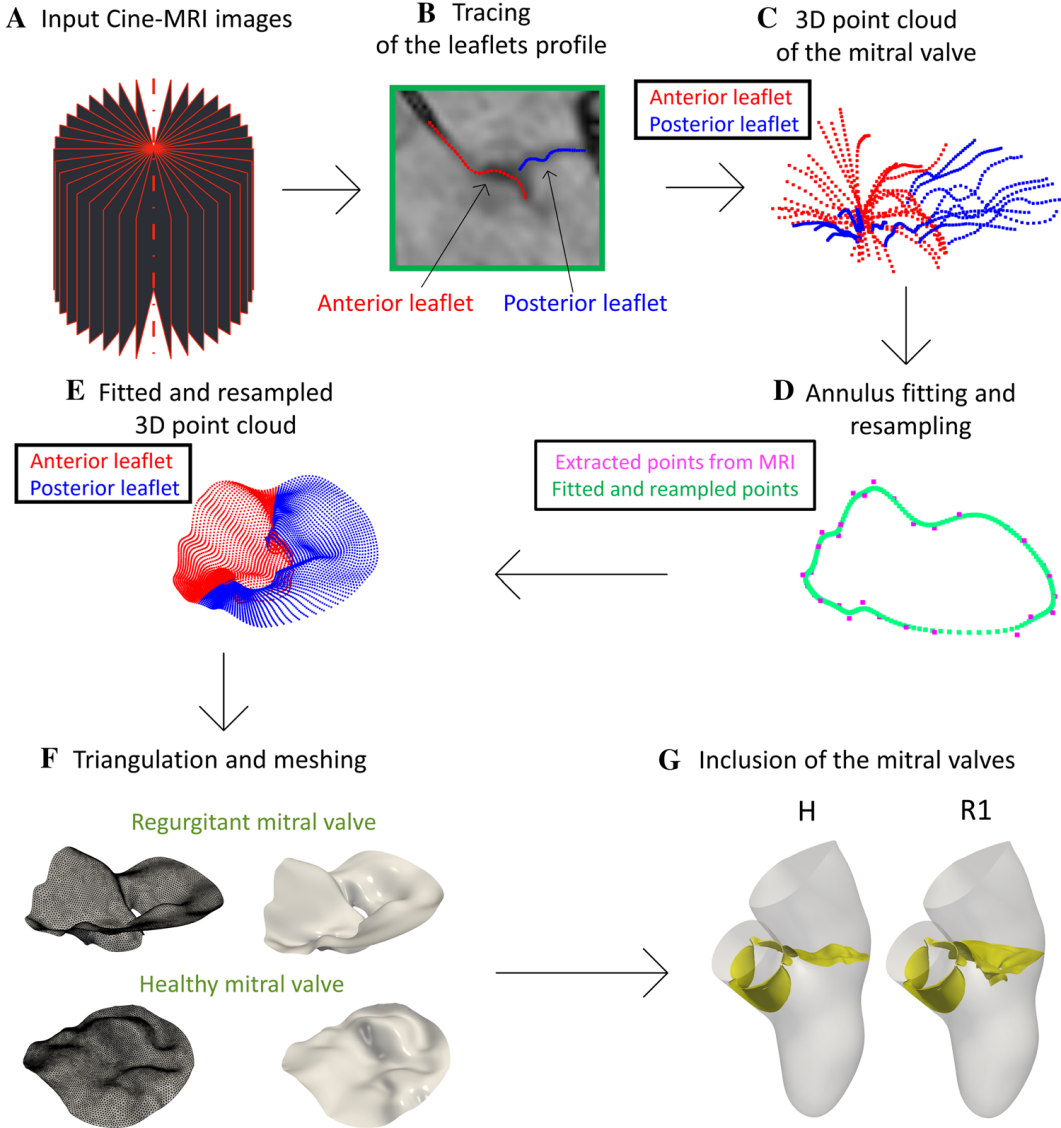
Flow chart to reconstruct the mitral valves and to define the virtual scenarios. (A) Cine-MRI images of the mitral valve. (B) Tracing of the leaflets profile on each of the 18 longitudinal planes. (C) 3D point cloud of the mitral valve obtained by tracing the leaflets profiles in all the planes. (D) Fitting and resampling procedure of the rings (the case of the annulus is reported here as an example). (E) The 3D point cloud as a result of the fitting and resampling procedure performed for all the rings. (F) Creation of a surface mesh of the mitral valve. (G) Generation of scenarios H and R1 (to obtain scenario R2, we dilated the geometry and the displacement field of R1 by a factor 1.25)

For the reconstruction of the mitral valves, we adapted to the systolic phase the method proposed in Ref. [67] for the diastolic case, see Fig. 2.

The starting point are Cine-MRI images of a healthy and of a regurgitant mitral valve acquired based on a protocol proposed for the first time in Ref. [67]. The operator performed a radial sampling by using 18 planes rotated every $10^{\circ}$ around the axis passing through the center of the annular plane and aligned with the left ventricle apex. The result of this protocol is reported in step A in Fig. 2 and represents the input data to reconstruct the mitral valve.

Then, such mitral images were imported in 3D Slicer (https://slicer.org/) and for each of the 18 acquired longitudinal planes we traced the leaflets profile in the ED configuration by using a spline curve sampled in 32 points (step B in Fig. 2). In the healthy case, in order to guarantee a full closure, we imposed that the positions of last points of the anterior and posterior leaflet profiles were equal.

We repeated this operation for all the 18 planes to obtain the final 3D point cloud representing the mitral valve profile (step C in Fig. 2). These data are stored in a matrix of cloud points composed by 1152 rows (32 sampling points $\times$ 36 leaflets profiles) $\times$ 3 columns (*x*, *y* and *z* coordinate). After, we imported this matrix in MATLAB (www.mathworks.com) to perform a smoothing process in order to reduce possible inaccuracies due to image noise and to the manual segmentation of the leaflets. To do this, we created a new matrix where blocks of 36 consecutive rows represent the (36) points of a ring (32 rings in total), with the first ring corresponding to the annulus and the last one to the free margin. Then, the points of each ring were fitted by using a B-Spline (see step D in Fig. 2). This resulted in a new 3D point cloud of 32,000 rows (1000 points $\times$ 32 rings) $\times$ 3 columns (*x*, *y* and *z* coordinate), see step E in Fig. 2. After, for each of the 3D point cloud of two mitral valves, a preliminary surface mesh was generated in MATLAB and then remeshed with a uniform edge length using MeshMixer (https://www.meshmixer.com), see step F in Fig. 2.

Finally, the mitral valves were added to the reconstructed geometry (Fig. 1) to build the three virtual scenarios under study (step G in Fig. 2). In particular, to guarantee a perfect adhesion between the mitral annulus and the ventricle, we placed the mitral valve in the valvular plane of the reconstructed geometry at the ES instant (see Fig. 1, step D) and calculated the minimum distance of the annulus with respect to the wall. After, we harmonically extended this distance over all the mitral valve by using the same harmonic extension algorithm used to extend the ventricular endocardial displacement. Finally, we warped the mitral valve according to such displacement (for the aortic valve we adopted the same strategy).

We point out that geometry and displacement field ${\mathbf {d}}^{i}_{{\text {h}},{\text {MRI}}}({\mathbf {x}})$ of H and R1 are the same at each of the six time instants (see Fig. 1, step D). Instead, to obtain the configuration of R2, in absence of patient-specific data, we dilated the geometry of R1 to reach a diameter of the left ventricle cavity equal to 7.2 cm; this represents a characteristic value of the maximum diameter of a dilated left ventricle cavity in presence of MVR according to Refs. [56, 65]. To do this, we expanded at each time frame the R1 geometry and the displacement field ${\mathbf {d}}^{i}_{{\text {h}},{\text {MRI}}}({\mathbf {x}})$ by a factor of 1.25. We employed the same dilation factor (1.25) for the whole geometry because of the difficulty to predict left atrium and aorta dilation. Indeed, they are dependent upon several variables, including, but not limited, to the degree and duration of MVR, the presence of associated lesions of the aortic valve and/or the ascending aorta, the presence and type of acute or chronic arrhythmias, the left ventricular function, the presence and degree of systemic hypertension, the utilization of medications to reduce pressure and/or volume overload [37, 25, 81, 12].

**Fig. 3:**
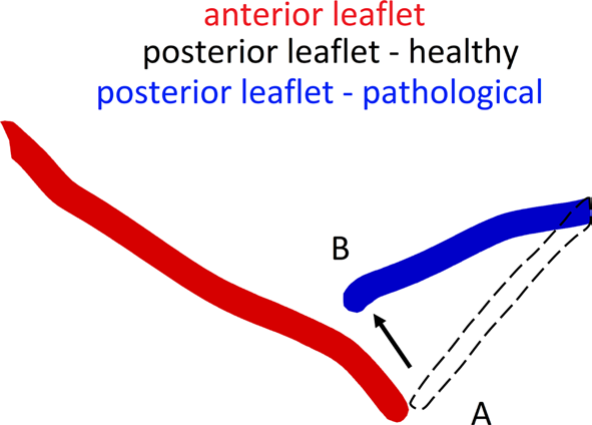
Section of the mitral valve with prolapse of the posterior leaflet. In red the anterior leaflet and in blue the posterior one. The dashed curve represents a possible position of the posterior leaflet in the healthy configuration. The prolapse of the P2 segment moves the posterior leaflet from configuration A to B towards the atrium

In Fig. 3, we showed a section of the regurgitant mitral valve (starting from the surface obtained at step F—above in Fig. 2). In red we reported the anterior leaflet and in blue the posterior one. As said, this valve has a posterior leaflet prolapse of the P2 segment, namely a displacement of the leaflet towards the left atrium [51]. The prolapse is emphasized by comparing the real pathological (prolapsed) posterior leaflet (B, in blue) with a possible configuration of the posterior leaflet in the healthy configuration (A, dashed line).

### Mathematical and Numerical Modeling

**Fig. 4:**
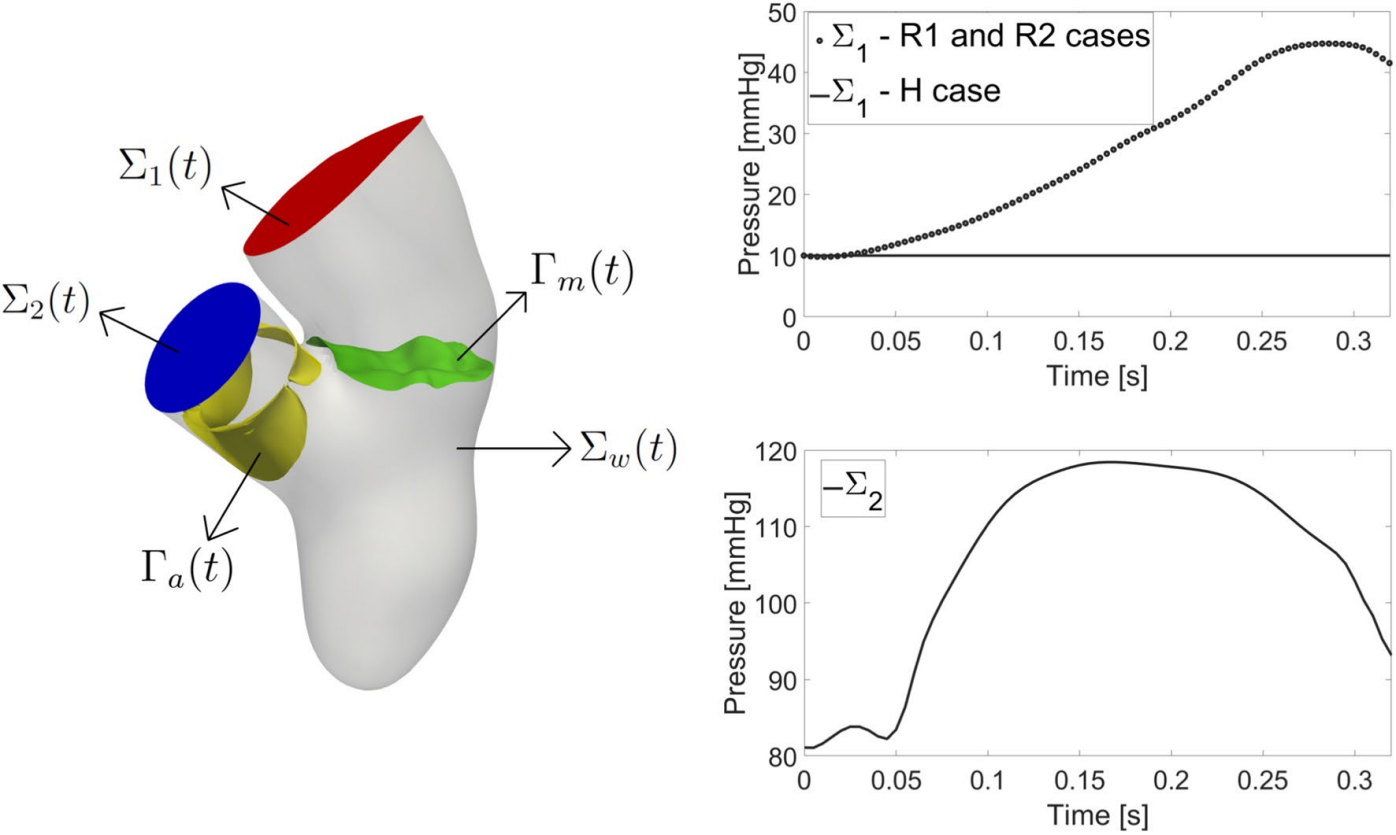
Left: computational domain $\Omega (t)$ with its boundaries: $\Sigma _{\text {w}}(t)$ represents the ventricular endocardium and the physical wall of the atrium and aortic root; $\Sigma _{1}(t)$ is the atrium outlet and $\Sigma _{2}(t)$ the aortic outlet. $\Gamma _{\text {a}}(t)$ is the surface of the aortic valve and $\Gamma _{\text {m}}(t)$ the surface of the mitral valve. Right: trend in time of the pressure imposed at the outlets $\Sigma _{1}(t)$ (up) and $\Sigma _{2}(t)$ (bottom) (for R1 the frequency has been suitably adapted)

Let $\Omega (t)$ be the computational moving domain of the complete geometry. The boundaries of $\Omega (t)$ are displayed in Fig. 4, left.

The motion of $\Omega$ is determined by the displacement field ${\mathbf {d}}_{{\text {h}},{\text {MRI}}}^{i}({\mathbf {x}})$ reconstructed and showed in Fig. 1. However, ${\mathbf {d}}_{{\text {h}},{\text {MRI}}}^{i}({\mathbf {x}})$ has been obtained only at the MRI acquisition times ($i=1,\ldots ,6$) and thus we employed a spline interpolation to obtain ${\mathbf {d}}_{{\text {h}}, {\text {MRI}}}({\mathbf {x}},t)$ for all *t* $\in$ [0, $T_{\text {S}}$].

We considered blood as an incompressible Newtonian fluid with density $\rho = 1.06\times 10^{3}$ kg/m$^{3}$ and dynamic viscosity $\mu = 3.5\times 10^{-3}$ Pa s. The systolic contraction of the complete geometry is taken into account by solving the fluid problem in an Arbitrary Lagrangian–Eulerian (ALE) formulation [78, 19]. The fluid domain is obtained by starting from the ventricular displacement ${\mathbf {d}}_{{\text {h}}, {\text {MRI}}}$ and by extending it into $\Omega$ through the solution of a linear elastic problem [85].

The presence of the valves is accounted by a resistive immersed method inspired by the one proposed in Ref. [36, 34]. In particular, we used the resistive immersed implicit surface (RIIS) method, whose main advantage is that the computational mesh and the surface of the valve do not need to be conforming [21]. Specifically, the RIIS method introduces, into the momentum balance of the Navier–Stokes equations, an additional term which penalizes the kinematic condition representing the adherence of the blood to the valves.

To capture the transition to turbulence [24], we used the large eddy simulation (LES) $\sigma$-model proposed for ventricular blood dynamics in Ref. [57] and successfully used in different hemodynamic applications [23, 2, 42]. In particular, this turbulence model is suited to handle wall bounded flows in complex geometries, such as ventricle and atrium [57].

In this framework, let index *n* indicate the approximation of quantities at time $t^{n}=n\ t$, where $\Delta t$ is the time discretization parameter. Then, with a first-order semi-implicit time discretization, the problem at time step $t^{n+1}$ reads:

1. Solve a linear elasticity problem to find the displacement ${{\mathbf {\widehat{d}}}}^{n+1}$ of the fluid domain^3^:
  1 $${\left\{ \begin{array}{ll} -\nabla \cdot \left[ 2\mu _{\text {EXT}}\nabla ^{\text {s}}{\mathbf {\widehat{d}}}^{n+1} + \lambda _{\text {EXT}}\left( \nabla \cdot {\mathbf {\widehat{d}}}^{n+1}\right) {\mathbf {I}}\right] = {\mathbf {0}} \quad &{} {\text {in}}\quad \widehat{\Omega }, \\ {{\mathbf {\widehat{d}}}}^{n+1} = \widehat{{\mathbf {d}}}_{{\text {h}}, {\text {MRI}}}^{n+1} \quad &{} {\text {on}} \quad {\widehat{\Sigma }}_{\text {w}}, \\ {\mathbf {\widehat{d}}}^{n+1} = {\mathbf {0}} \quad &{} {\text {on}} \quad {\widehat{\Sigma }}_{1},\\ \left[ 2\mu _{\text {EXT}}\nabla ^{s}{\mathbf {\widehat{d}}}^{n+1} + \lambda _{\text {EXT}}\left( \nabla \cdot {\mathbf {\widehat{d}}}^{n+1}\right) {\mathbf {I}}\right] \cdot {\widehat{\mathbf {n}}} = {\mathbf {0}} \quad &{} {\text {on}} \quad {\widehat{\Sigma }}_{2}, \end{array}\right. }$$
  with $\nabla ^{\text {s}}$ the symmetric gradient and ${\mathbf {I}}$ the identity matrix. $\mu _{\text {EXT}}$ and $\lambda _{\text {EXT}}$ were both set to be the value of 0.4 Pa to avoid mesh elements degeneration during the deformation. Notice the homogeneous Dirichlet condition on $\Sigma _{1}$, since we want to keep fixed the atrial section, and the homogeneous Neumann condition on $\Sigma _{2}$ in order to let it free to move in accordance with the rest of the geometry.
2. Update the fluid domain $\Omega ^{n+1}$ = $\widehat{\Omega }$ + ${\mathbf {\widehat{d}}}^{n+1}$ and calculate the fluid domain velocity ${\widehat{{\mathbf {u}}}}^{n+1}_{\text {ALE}}$ = $({\mathbf {\widehat{d}}}^{n+1} - {\mathbf {\widehat{d}}}^{n})/\Delta t$;
3. Compute the wall velocity ${\widehat{{\mathbf {u}}}}_{{\text {h}}, {\text {MRI}}}^{n+1} = (\widehat{{\mathbf {d}}}_{{\text {h}}, {\text {MRI}}}^{n+1}-\widehat{{\mathbf {d}}}_{{\text {h}}, {\text {MRI}}}^{n})/\Delta t$;
4. Solve the ALE Navier–Stokes equations in the known domain $\Omega ^{n+1}$ to find the pressure $p^{n+1}$ and the blood velocity ${\mathbf {u}}^{n+1}$^4^:
  2 $${\left\{ \begin{array}{ll} \rho \dfrac{{\mathbf {u}}^{n+1}-{\mathbf {u}}^{n} }{\Delta t} + \rho \left( {\mathbf {u}}^{n} - {\mathbf {u}}^{n+1}_{\text {ALE}}\right) \cdot \nabla {\mathbf {u}}^{n+1} \\ \quad - \nabla \cdot \left( 2(\mu _{\text {sgs}}({\mathbf {u}}^{n})+\mu ){\mathbf {D}}({\mathbf {u}}^{n+1})\right) + \nabla p^{n+1} \\ \quad + \sum _{i=a,m} \dfrac{R}{\varepsilon }\left( {\mathbf {u}}^{n+1}- {\mathbf {u}}^{n+1}_{\Gamma _{i}}\right) \delta _{\Gamma _{i}, \varepsilon _{i}} = {\mathbf {0}}\, \ &{} {\text {in}}\ \Omega ^{n+1}, \\ \nabla \cdot {\mathbf {u}}^{n+1} = 0 \ &{} {\text {in}}\ \Omega ^{n+1},\\ {\mathbf {u}}^{n+1} = {\widehat{{\mathbf {u}}}}_{{\text {h}}, {\text {MRI}}}^{n+1} &{} {\text {on}}\ \Sigma ^{n+1}_{\text {w}},\\ \end{array}\right. }$$
  with a null initial condition in $\Omega ^{0}$.

The convective term in the momentum equation is treated in a semi-implicit way. The velocity strain rate tensor ${\mathbf {D}}$(${\mathbf {u}}^{n+1}$) is defined by ${\mathbf {D}}({\mathbf {v}}) = \left( \nabla {\mathbf {v}}+ (\nabla {\mathbf {v}})^{\text {T}}\right) /2$. The sub-grid viscosity $\mu _{\text {sgs}}$ of the $\sigma$-model is given by

$\mu _{\text {sgs}} = \rho C\Delta ^{2} \sigma _{3}(\sigma _{1} - \sigma _{2})(\sigma _{2}-\sigma _{3})/\sigma _{1}^{2}$, with $\sigma _{1}({\mathbf {x}})> \sigma _{2}({\mathbf {x}}) > \sigma _{3}({\mathbf {x}})$ the singular values of $\nabla {\mathbf {u}}^{n}$. The average mesh element size $\Delta$ is equal to 1.5 mm for scenario H and R1 and 1.9 mm for R2 while C is a constant set to the value of 1.5 [42, 2]. Notice the explicit treatment of the LES non-linearity.

In the RIIS model, *R* is the resistance coefficient, whereas $\varepsilon$ is half of the leaflets thickness. For both the mitral and aortic valves, we set $R=10^{4}$ kg/m s, a penalization large value used to enforce the kinematic constraint [29], and $\varepsilon =0.75$ mm. The prescribed leaflets velocity ${\mathbf {u}}^{n+1}_{\Gamma _{i}},\,i=m,a,$ is zero for the mitral valve, since we considered only the closed configuration, and for the aortic valve, since the opening/closure mechanism (occurring only in the regurgitant cases R1 and R2) was modeled in an on/off modality, by considering the two geometric configurations (open/closed) of the aortic valve template. The opening phase (modeled for R1 and R2, see “Inclusion of Left Atrium, Aortic Valve, and Aorta” section) was instantaneous and triggered by a positive pressure jump between the ventricle and the aorta, whereas the closure occurred when a negative flow rate developed at the aortic valve plane [36, 55]. Furthermore, in order to guarantee a perfect adhesion between the valves and the walls at each time instant, we imposed both the valves to move in accordance with the ALE displacement.^5^

Finally, we have that $\delta _{\Gamma _{i}, \varepsilon _{i}},\,i=m,a,$ is a smoothed Dirac delta function representing a layer, with thickness $2\varepsilon$, around the surface of the valve, given by the following expression [21, 29]:

3 $$\delta _{\Gamma _{i}, \varepsilon _{i}}(\varphi _{i}) = {\left\{ \begin{array}{ll} \dfrac{1+\cos (\pi \varphi _{i}/\varepsilon _{i})}{2\varepsilon _{i}}\quad &{}{\text {if}} \quad |\varphi _{i} |\le \varepsilon _{i}, \\ 0 \quad &{}{\text {if}} \quad |\varphi _{i} |>\varepsilon _{i}, \end{array}\right. }$$

where $\varphi _{i}$ is a signed distance function that implicitly describes the *i*th immersed surface $\Gamma _{i}$ as $\Gamma _{i}$ = {${\mathbf {x}}$ : $\varphi _{i}$(${\mathbf {x}}$) = 0}.

Regarding the boundary conditions for problem (2), at the atrial outlet $\Sigma _{1}$, we prescribed a constant pressure of 10 mmHg taken from the Wiggers diagram [58] for scenario H, and a time dependent pressure taken from Refs. [35, 75] for the regurgitant scenarios R1 and R2 (Fig. 4, right). In the latter cases, the authors observed that the formation of a RV in the atrium results in a marked increase of the atrial pressure (elevated V-wave). Nevertheless, in absence of any information about the range of validity of the pressure waveform provided in Refs. [35, 75], we applied it to both R1 and R2 cases. In addition, in scenarios R1 and R2, to avoid possible backflows instabilities, we imposed null tangential velocity [50]. At the aorta outlet $\Sigma _{2}$ we imposed a time dependent physiological aortic pressure taken from Ref. [35] for all the scenarios (Fig. 4, right) because in presence of compensated MVR, the heart is able to guarantee a physiological afterload [15, 60]. Finally, a no-slip condition is imposed on $\Sigma _{\text {w}}$ in (2), prescribing the wall velocity coming from imaging.

To solve numerically the Eqs. (1) and (2), we considered first-order finite elements. Equation (2) was stabilized by means of a Streamline Upwind Petrov–Galerkin/Pressure-Stabilizing Petrov–Galerkin (SUPG/PSPG) scheme [63, 43]. We used the multiphysics high performance library $life^{x}$ [39] (https://lifex.gitlab.io/) based on the deal.II core [66] and developed in the iHEART project (https://iheart.polimi.it/).

Hexahedral meshes were generated by using suitable algorithms developed in VMTK, in particular, for the mesh generation based on *TetGen* (*vmtkmeshgenerator*) and for the mesh refinement (*vmtkmeshrefinement*) [46, 61]. The average mesh element size was equal to 1.5 mm (1.9 mm) with a local refinement of 0.4 mm (0.35 mm) close to the mitral valve for H and R1 (R2). These values were chosen after a mesh convergence analysis performed on scenario H, which showed differences of the quantities of interest of at most 2% when the mesh was refined by a factor 10% (see “Appendix”). Regarding the time discretization parameter, we set $\Delta t = 2.5\times 10^{-4}s$, a value chosen after a convergence analysis showing that halving this value the quantities of interest did not change within a discrepancy of 2% (see “Appendix”).

Numerical simulations were run on the cluster iHEART (Lenovo SR950 8 x 24-Core Intel Xeon Platinum 8160, 2100 MHz and 1.7TB RAM) available at MOX, Dipartimento di Matematica, Politecnico di Milano.

## Numerical Results

**Fig. 5:**
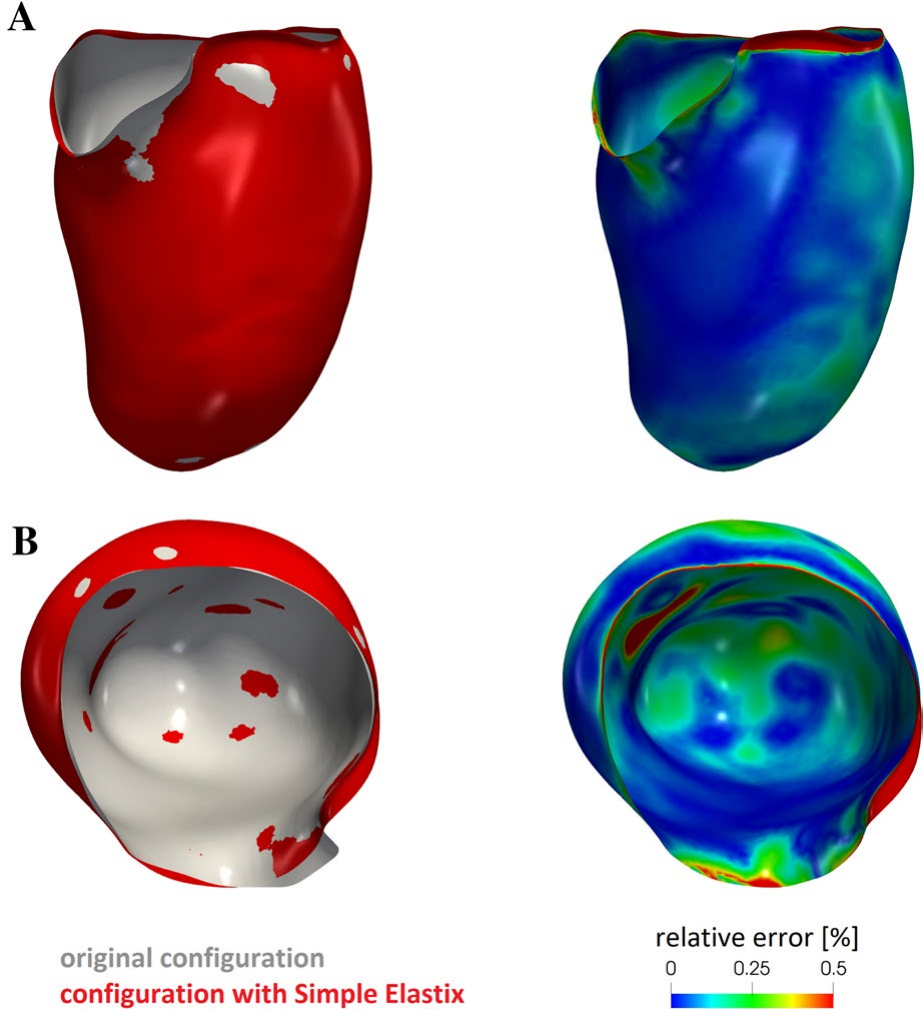
For each of the two views **A** and **B** comparison between the original end diastolic reconstructed surface and the one obtained after the registration procedure (left) and the corresponding relative error (right)

We start our analysis by reporting the results in terms of geometry reconstruction in order to check the validity of the registration procedure, see “Description of the Scenarios” section. In particular, in Fig. 5, left, we reported for the ED frame the original surface obtained from MITK and that obtained by warping the reference configuration (i.e. the end systolic one) by the corresponding computed displacement field (we chose the ED surface because it is characterized in principle by the largest errors). These results underlined an excellent agreement between the two surfaces as confirmed by the relative error of pointwise position, see Fig. 5, right, whose average value is 0.16%.

**Fig. 6:**
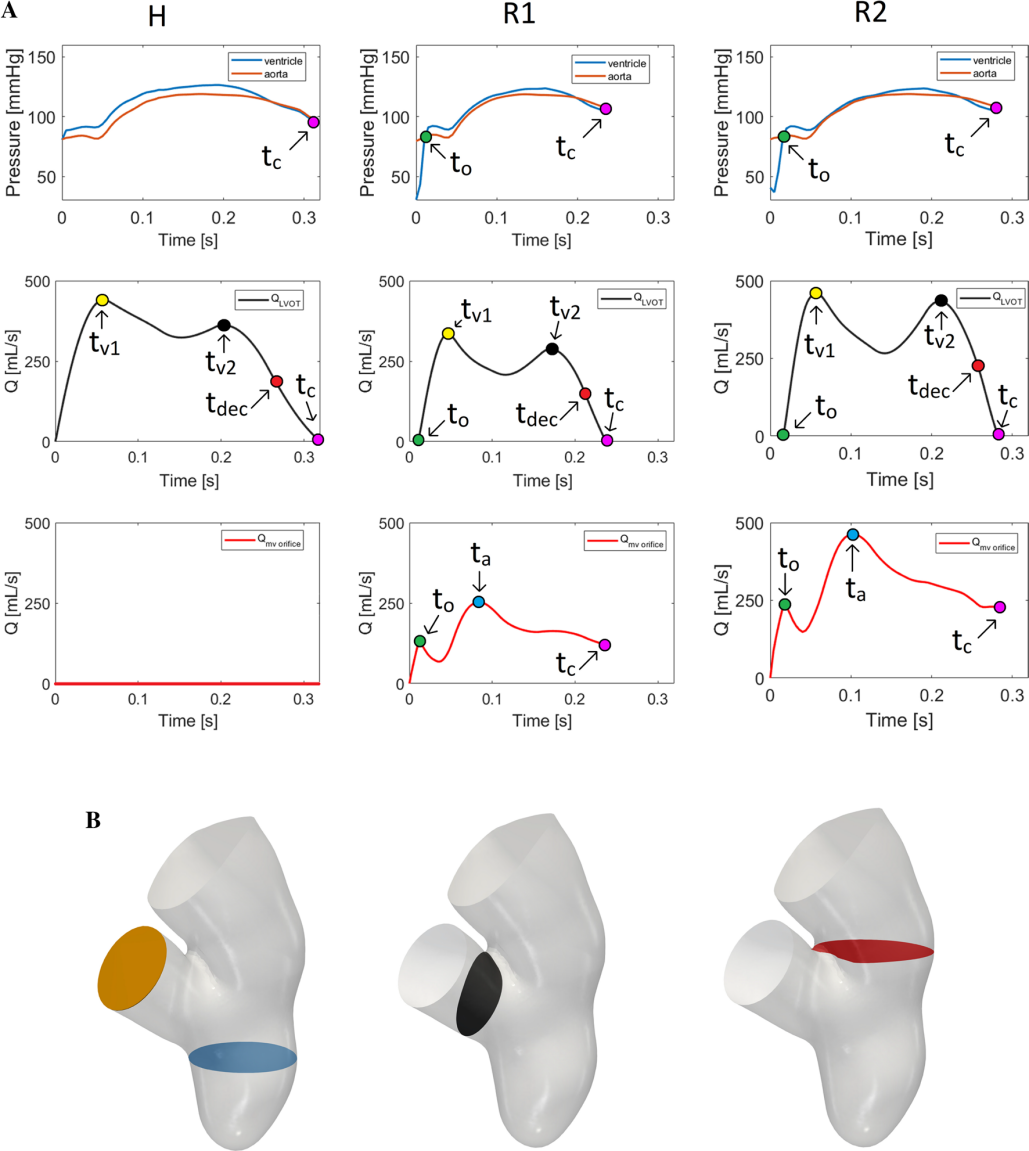
Panel **A**, top: trend in time of the ventricle and aortic pressures (computed in the blue and orange slices reported in panel **B**, left); $t_{\text {o}}$ is the time instant where the aortic valve opens; $t_{\text {c}}$ is the time instant where the aortic valve closes. Panel **A**, middle: plot over the time of the flow rate at the level of the LVOT (computed in the black slice reported in panel **B**, middle); $t_{{\text {v}}1}$ and $t_{{\text {v}}2}$ are the peak instants and $t_{\text {dec}}$ the instant of middle deceleration. Panel **A**, bottom: plot over the time of the flow rate at the level of the mitral valve orifice (computed in the red slice reported in panel **B**, right); $t_{\text {a}}$ is the time instant of the maximum flow rate. Panel **B** sections of interest

Then, we discuss the computational opening of the aortic valve. As stressed in “Geometric Reconstruction of the Left Ventricular Endocardium” section, for scenario H we could not simulate the isovolumic contraction, thus we began our simulation at the instant $t_{\text {o}}=0$ s just after the opening of the aortic valve. Instead, for R1 and R2 we observed that the pressure of the left ventricle was lower than the aortic one for $t=0$ s, due to mitral regurgitation, see Fig. 6A, top (ventricle and aortic pressures were calculated on slices showed in Fig. 6B, left). In particular, the opening of the aortic valve occurred at $t_{\text {o}} = 0.010$ s and at $t_{\text {o}} = 0.016$ s for R1 and R2, respectively.

Figure 6A, top, also showed slightly larger values of the ventricular-aortic pressure drop for H with respect to R1 and R2, that featured very similar behaviours. This was also confirmed by the values reported in Table 1 where we computed the average in time (from $t_{\text {o}}$ to the closure instant $t_{\text {c}}$) of the pressure drop $\overline{\Delta P}$ between the two slices of Fig. 6B, left.

**Table 1:** Values of the quantities of interest computed for the three scenarios

Scenario	$\overline{\Delta P}$ (mmHg)	$t_{\text {c}}-t_{\text {o}}$ (s)	SV (mL)	CO (L/min)	RV (mL)	RF (%)	$U_{\text {AV}}$ (m/s)	$U_{\text {MV}}$ (m/s)	$\overline{R}$ (–)	$\overline{{\text {WSS}}_{\text {MV}}}$ (Pa)	$\overline{{\text {WSS}}_{\text {W}}}$ (Pa)
H	5.5	0.32	92	6.9	0	0	1.8	0.0	0.1	0.3	0.1
R1	2.6	0.22	50	4.5	40	44	1.3	6.5	2.4	5.4	2.9
R2	2.0	0.26	85	6.4	89	51	0.9	5.3	3.2	3.9	3.0

$\overline{\Delta P}$, pressure drop between ventricle and aorta; $t_{\text {c}} - t_{\text {o}},$ duration of the aortic ejection phase; *SV*,  stroke volume; *CO*,  cardiac output; *RV*,  regurgitant volume; *RF*,  regurgitant fraction; $U_{\text {AV}}$ and $U_{\text {MV}},$ maximum velocity magnitude at the aortic valve and trough the mitral valve orifice; $\overline{R},$ time average of the ratio between sub-grid and physical viscosity in the atrium; $\overline{{\text {WSS}}_{\text {MV}}}$ and $\overline{{\text {WSS}}_{\text {W}}},$ time average of the trend in time of the WSS magnitude acting on selected regions of the mitral valve and of the atrial walls

**Fig. 7:**
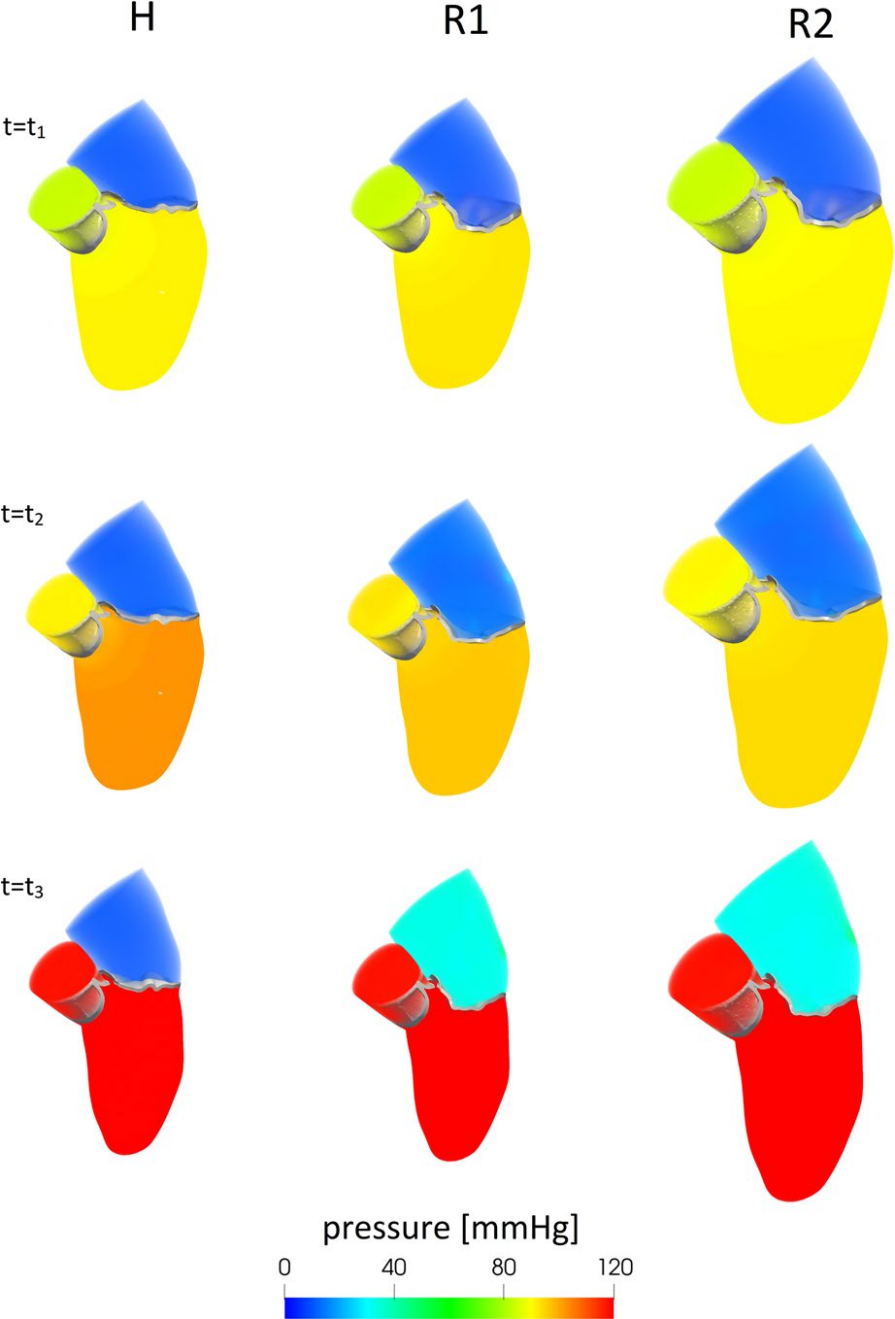
Pressure field at three representative time instants, $t_{1}$, $t_{2}$ and $t_{3}$ (see the text for their definition), in the three scenarios

To obtain a description of the spatial distribution of the pressure, in Fig. 7, we reported the pressure field in the whole domain at three representative instants $t_{1}$, $t_{2}$ and $t_{3}$. In particular, for H and R2 $t_{1} = 0.020$ s, $t_{2} = 0.060$ s, and $t_{3} = 0.200$ s, which correspond for R1 to $t_{1} = 0.017$ s, $t_{2} = 0.051$ s, and $t_{3} = 0.170$ s. We noticed that in all the scenarios and at all the three reported instants, the ventricular and atrial pressure was homogeneous in space. In particular, the ventricular pressure (as confirmed by Fig. 6A, top) was almost the same for the three scenarios, whereas the atrial one was (according to the outflow boundary conditions) much larger for the regurgitant cases.

Regarding the closure of the aortic valve, from Fig. 6A we noticed that the closing stage of the aortic valve in H occurred at $t_{\text {c}} = 0.32\,{\text {s}}$, while in R1 and R2 much earlier, i.e. at $t_{\text {c}} = 0.24\ {\text {s}}$ and at $t_{\text {c}} = 0.28\ {\text {s}}$, respectively. In Table 1, we reported the difference between $t_{\text {c}}$ and $t_{\text {o}}$, representing the effective duration of the aortic ejection phase.

In Fig. 6A, middle, we reported the trend in time of the flow rate evaluated at the level of the LVOT (Fig. 6B, middle). For all the three curves, we could recognize two significant peaks, at $t_{{\text {v}}1}$ (yellow points) and at $t_{{\text {v}}2}$ (black points) and a final phase of deceleration.

In Fig. 6A, bottom, we reported the trend in time of the flow rate evaluated at the level of the mitral valve orifice (Fig. 6B, right). For R1 and R2, at $t_{\text {o}}$ we had a local maximum due to the sudden opening of the aortic valve. After, both the curves started again to increase, reaching the global maximum at $t_{\text {a}}$ (blue points) that was higher for R2.

From these curves we calculated some cardiac indices reported in Table 1: the SV and the regurgitant volume (RV), obtained by integrating in time the flow rates evaluated at the level of the LVOT and through the mitral orifice, respectively; the CO, given by the product between SV and the heart rate and the RF defined as the ratio between RV and the sum of the SV and RV. The value of the SV of the healthy case was compared with the one calculated in the preprocessing phase (i.e. by processing only Cine-MRI images), finding for the latter a value ${\text {SV}}_{\text {PP}} = 93.1\ {\text {mL}}$. The comparison suggested the presence of a small amount of volume across the valve equal to 1.1 mL, corresponding to $1.21\%$ of the total volume. This highlights how the RIIS method led to very small lacks of volume.

**Fig. 8:**
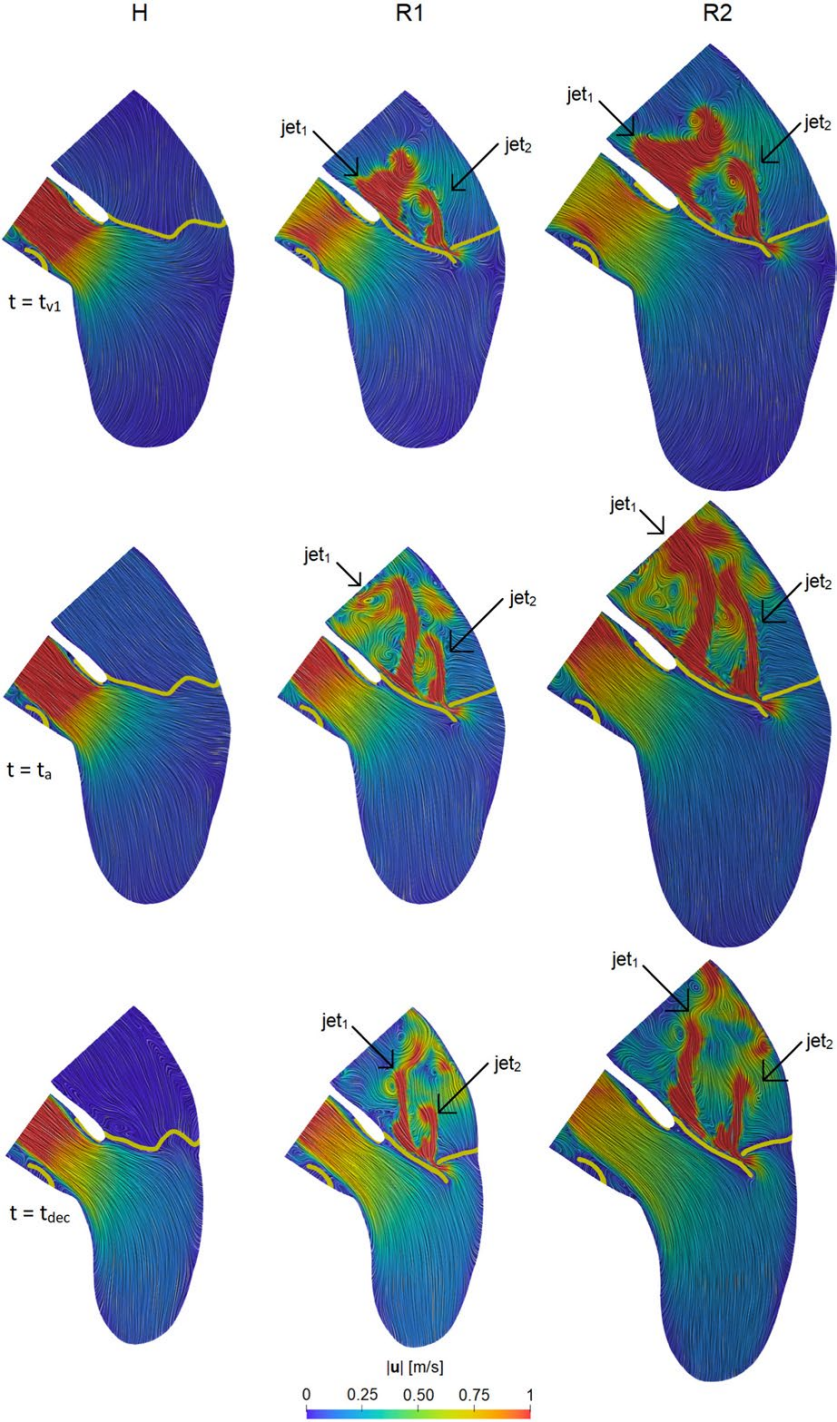
Velocity magnitude at three time instants in the three scenarios. In yellow we reported a slice of the two valves. For the instants refer to Fig. 6A. For H we used the value of $t_{\text {a}}$ taken from R2

To obtain a description of the the velocity patterns in the whole domain, in Fig. 8, the blood velocity field was represented on a 2D longitudinal slice to show the hemodynamics in the three scenarios.

In particular, we considered three representative time instants: $t_{{\text {v}}1}$, $t_{\text {a}}$ ($t_{\text {a}}$ for H being the same as R2) and the instant of middle deceleration of the ventricle flow rate, $t_{\text {dec}} = \frac{t_{{\text {v}}2} + t_{\text {c}}}{2}$, see Fig. 6A. We observed that, as expected, in scenario H all the blood flowed in the aorta; instead, we noticed the formation of a *regurgitant jet* in the atrium for R1 and R2. At $t_{{\text {v}}1}$, in both the regurgitant cases the global velocity pattern was similar; the jet split in two different structures: one developing along the anterior leaflet (${\text {jet}}_{1}$) and the other one arising directly from the free margin (${\text {jet}}_{2}$) with a straighter direction. At $t_{\text {a}}$, also ${\text {jet}}_{1}$ assumed a straight configuration; in particular, in R2 the two jets collided close to the atrium outlet. In both R1 and R2 we also noticed that the velocities were elevated close to the wall of the atrium and that backflows formed at the outlet. At $t_{\text {dec}}$, the two jets split up in both the scenarios and, in correspondence of the atrial outlet, we noticed some fluctuations of the jets. In Table 1, we reported the maximum velocity magnitude $U_{\text {AV}}$ and $U_{\text {MV}}$ at the aortic valve plane and through the mitral valve orifice, respectively.

**Fig. 9:**
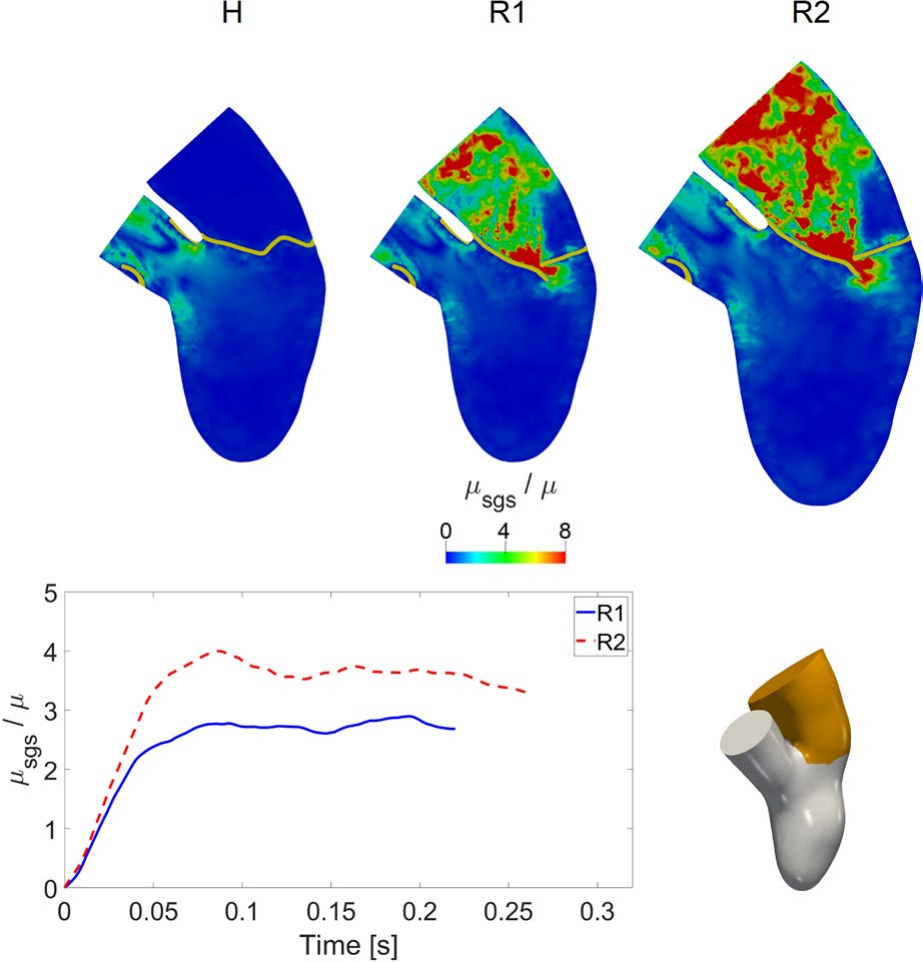
Top: ratio between the turbulent viscosity $\mu _{\text {sgs}}$ and the physical viscosity $\mu$ in the three scenarios at $t_{\text {a}}$. For H we used the value of $t_{\text {a}}$ taken from R2. Bottom: trend in time of average (in space) in the atrium of the ratio between the turbulent viscosity $\mu _{\text {sgs}}$ and the physical viscosity $\mu$ for the regurgitant scenarios R1 and R2

In Fig. 9, top, the ratio $\mu _{\text {sgs}}/\mu$ between turbulent and physical viscosity was reported in the three cases at $t_{\text {a}}$.

From these results, we found elevated values of $\mu _{\text {sgs}}$ for R1 and R2 in the atrium, in particular in the regions with high velocities (see Fig. 8): at the level of the mitral valve orifice, where the sub-grid viscosity reached values also eight times greater than the value of the physical viscosity, and in the middle of the atrium, where chaotic and irregular velocity patterns were noticed. Similar patterns were found for the other time instants. Instead, as expected, scenario H did not feature transition to turbulence. In Fig. 9, bottom, we reported the trend in time of the average in the atrium of the ratio $\mu _{\text {sgs}}/\mu$ for the regurgitant scenarios. In R2 we noticed a higher formation of turbulent viscosity as confirmed by Table 1, where we reported the average in time $\overline{R}$ of $\mu _{\text {sgs}}/\mu$, confirming the essential absence of transition to turbulence in the H case.

**Fig. 10:**
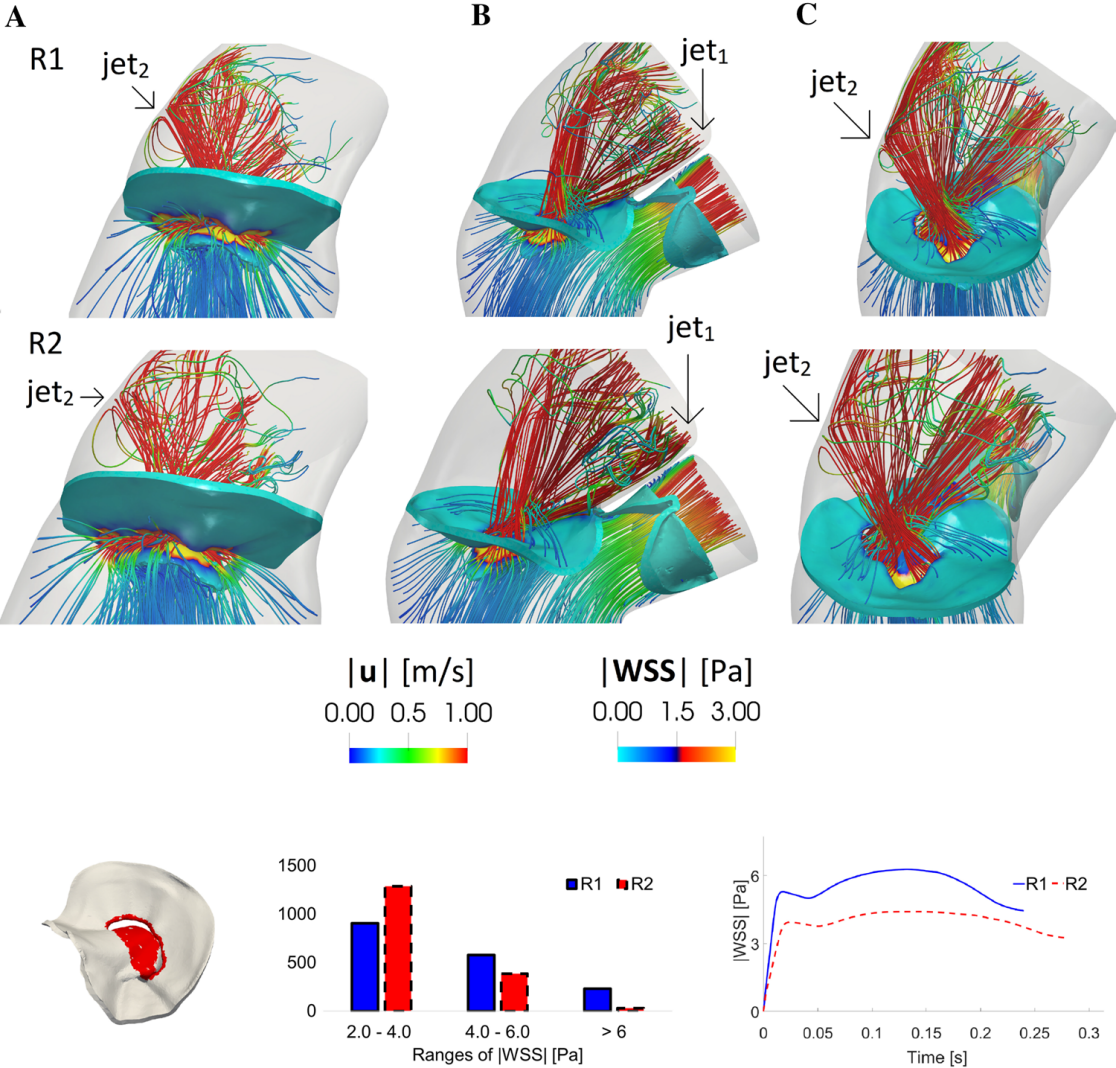
Top: streamlines of the velocity magnitude through the mitral and aortic orifices and magnitude of the WSS on the mitral valve. Instant $t_{\text {a}}$; three different views; R1 and R2 configurations. Bottom, left: in red the selected region with the points used to compute the histogram and the plot reported on the right. Bottom, middle: distribution of the WSS magnitude acting on the red region at $t_{\text {a}}$. Bottom, right: trend in time of the average (in space) of the WSS magnitude acting on the red region

In Fig. 10, top, we reported the streamlines through the mitral and aortic orifices and the magnitude of the WSSs on the mitral valve at instant $t_{\text {a}}$, for the regurgitant scenarios in three different views, namely A, B and C.

We noticed a hotspot of shear forces localized at the mitral orifice, where high velocities occurred (see also Fig. 8). We also observed, as expected, a chaotic regurgitant flow downstream the valve orifice, with swirling structures filling the atrium. Although the velocity patterns were very similar, these chaotic structures were more evident in R2 due to the higher values of the flow rate. In particular, we noticed the presence of the two regurgitant jets ${\text {jet}}_{1}$ (developing along the anterior leaflet, view B) and ${\text {jet}}_{2}$ (impinging against the atrial walls, views A and C).

In Fig. 10, bottom, we reported a histogram showing the distribution of the WSS magnitude at $t_{\text {a}}$, clustered in three intervals, acting on the red region reported on the left. This region was selected as representative of elevated WSS and corresponds to the free margin and to a part of the anterior leaflet. We noticed that, even if the majority of the values fell in the range 2–4 Pa, more elevated values ($> 6\ {\text {Pa}}$) were found for a significant number of points, especially for R1. We also reported the average of the WSS in the red region as a function of time. We observed that R1 featured higher WSS values than R2. This was also confirmed by the values reported in Table 1, where we computed the corresponding average in time $\overline{{\text {WSS}}_{\text {MV}}}$.

**Fig. 11:**
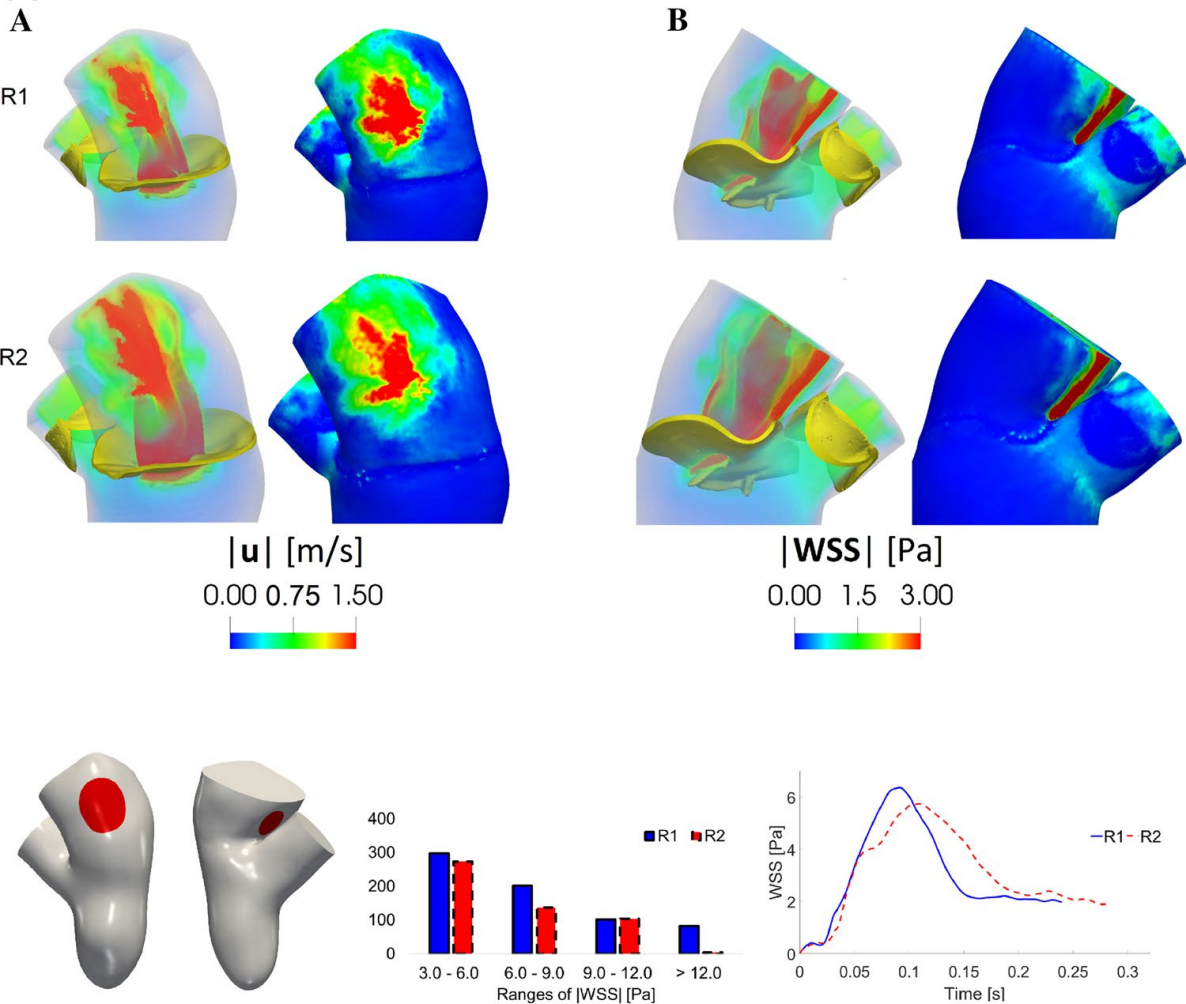
Top: for each of the columns **A** and **B**: volume rendering of the velocity magnitude (left) and spatial distribution of the WSS magnitude (right). Time $t_{\text {a}}$. Bottom, left: in red the selected regions with the points used to compute the histogram and the plot on the right. Bottom, middle: distribution of the WSS magnitude acting on the red regions at $t_{\text {a}}$. Bottom, right: trend in time of the average (in space) of the WSS magnitude acting on the red regions

In Fig. 11, top, we reported, for the regurgitant scenarios R1 and R2, the volume rendering of the velocity magnitude and the spatial distribution of the WSS magnitude at instant $t_{\text {a}}$, evaluated in the atrial region in two different perspectives.

From this figure we observed that in R1 and R2 the combined action of the two regurgitant jets ${\text {jet}}_{1}$ and ${\text {jet}}_{2}$ gave rise to two different zones of high WSS: the area where ${\text {jet}}_{2}$ impinged against the wall of the atrium (view A) and the area where ${\text {jet}}_{1}$ rubbed against the wall of the atrium adjacent to the anterior leaflet (view B, refer also to Figs. 8 and 10). In Fig. 11, bottom, we reported a histogram showing the distribution of the WSS magnitude at $t_{\text {a}}$, clustered in four intervals, acting on the two red regions reported on the left. These regions were selected as representative of elevated WSS and correspond to the areas where the regurgitant jets impinged against the atrial wall, see Fig. 11, top. We noticed that, even if the majority of the values fell in the range 3–6 Pa, more elevated values of the WSS (up to 12 Pa, or even more for R1) were found for a significant number of points. All these ranges resulted much higher than those of H where all the values fell in the interval 0.1–0.5 Pa. We also reported the average of the WSS in the two red regions as a function of time. In Table 1, we computed the corresponding average in time $\overline{{\text {WSS}}_{\text {W}}}$ and the values were very similar for R1 and R2 and much higher with respect to H.

## Discussion

In this work we performed an image-based computational study to analyze the hemodynamics in presence of a healthy (H) and regurgitant valve with either increased heart rate (R1) or dilated left ventricle (R2). These two phases represent two different mechanisms the heart employs to compensate the decreasing of the CO due to MVR. This process is known as *heart remodeling*, however what is still unclear in the literature is the interplay between all the factors involved (such as pressure, velocity, volumes and shear forces) [59].

First, analysing the ventricular-aortic pressure drop which represents and quantifies the force generated by the ventricle to win the aortic resistances, we found that in R1 and R2 the ventricle was not able to provide the same pressure drop between ventricle and aorta of the H case (Figs. 6A, top, 7 and values of $\overline{\Delta P}$ in Table 1). The consequence of lower values of pressure drop in the regurgitant scenarios was the shortening of the aortic ejection phase interval $t_{\text {c}}-t_{\text {o}}$, with a delay of the opening of the aortic valve at $t_{\text {o}}$ and an advance of the closing time $t_{\text {c}}$ (see Table 1). This time interval could be of interest for a better knowledge of the pathology and also useful from the clinical point of view, since it may help the physician in the quantification of the degree of MVR, as done in some clinical studies [1]. We also observed from Fig. 7 almost the same ventricular pressure among the three scenarios, despite the increased atrial pressure in the regurgitant cases. This suggested that the ventricular pressure was mainly driven by the wall displacement (which indeed was the same for the three scenarios) rather than by the health state of the mitral valve.

Second, as for the ventricular-atrial pressure drop, we found that it was the same for the two regurgitant cases (see Fig. 7), suggesting that it is mainly determined by the shape and dimension of the regurgitant orifice rather than by the overall conditions (heart rate, dilation,...).

Regarding our choice of opening instantaneously the aortic valve in the regurgitant scenarios, we observed that when it opened, the blood started to be ejected into the aorta with a force that is proportional to the positive pressure drop between the ventricle and aorta, but anyway too small to lead to potentially unphysiological values of the velocity. This is confirmed by Fig. 6A, left and middle, where we noticed, at the first time instants of the aortic valve opening, physiological values and trends of the ventricular pressure and flow rate, suggesting that the instantaneously opening of the aortic valve did not have so much influence on the blood velocity and pressure.

In scenario H all the blood was ejected into the aorta with a flow peak at $t_{{\text {v}}1}$ of 440 mL/s and a maximum ventricle pressure of 126.5 mmHg; these values were consistent with standard physiological data [49, 68]. Instead, in R1 and R2 part of the blood flowed back to the atrium. In all the scenarios, the ventricular flow rate featured two peaks (Fig. 6A, middle). This specific behavior was previously found in other studies [35, 9, 30].

From the flow rates through the aortic and mitral valve we calculated some cardiac indices (see Table 1), used by the physicians to elaborate the diagnosis. In R1 and R2 we noticed that such indices fell into the physiological ranges [20] despite the reduced CO with respect to H, highlighting the ability of the system to compensate for MVR by increasing the heart rate and/or augmenting the ventricular dimensions. In particular, in R1 the increasing of the heart rate provided more blood flow to aorta than to left atrium (regurgitant fraction ${\text {RF}} < 50\%$). Instead, in R2 the ventricle dilation allowed to sustain a physiological value of SV, even if the volume of blood returning to the left atrium was larger (${\text {RF}}> 50\%$).

Regarding blood velocity, in scenario H the maximum velocity magnitude $U_{\text {AV}}$ through the aortic valve was 1.8 m/s, a value comparable with those found in other studies [6, 82], while in the regurgitant scenarios the velocity values decreased to 1.3 and 0.9 m/s for R1 and R2, respectively. These values shown a correlation with the values of RV and RF reported in Table 1, in particular, larger values of regurgitation indices were characterized by smaller values of $U_{\text {AV}}$. This is in accordance with the decreased flow rate through the aortic valve in presence of MVR. Notice, however, that for R2 $U_{\text {AV}}$ is smaller than R1, despite the aortic flow rate, due to the dilation, is larger (see Fig. 6, middle, right).

Moreover, in R1 and R2 we found chaotic velocity patterns in the atrium (Fig. 10) with values of 6.5 and 5.3 m/s for the regurgitant maximum velocity $U_{\text {MV}}$ trough the mitral valve, respectively. These values were similar to others found in literature [89, 64, 70]. Interestingly, we noticed that $U_{\text {MV}}$ was higher in R1, despite lower values of RV and RF than R2. This apparently counter-intuitive behavior is due to the smaller mitral orifice regurgitant area experienced by the blood flow in R1. Furthermore, in both scenarios, the regurgitant jet split in two different structures, one of which (${\text {jet}}_{1}$) developed along the anterior leaflet (see Figs. 8 and 10). This behavior was in accordance with the Carpentier’s functional classification [14, 45, 72], intensively used by cardiologists for diagnosis purposes, for which, in case of MVR due to a prolapse, the jet is directed away from the pathological leaflet, in our case the posterior one [72].

We also noticed that, in the regurgitant cases, the velocity patterns in the atrium were comparable, due to the similar shape of the mitral orifices, despite their different size. Thus, the dilation of the geometry did not affect the distribution and the direction of the retrograde flow leading to an impingement of blood against the atrial wall in the same areas.

The formation of the regurgitant jet in the atrium led to high values of the subgrid viscosity in the turbulence model (Fig. 9 and turbulence index $\overline{R}$ in Table 1) suggesting that the formation of transition to turbulence in the left atrium could occur during systole in the regurgitant cases, especially in the dilated scenario.

MVR also yielded to high values of WSS on the mitral valve and on the atrial wall (see Figs. 10, 11 and Table 1), especially if compared with the values computed for the healthy case H and with the physiological range (0.4–1.2 Pa) found in the literature [52, 80]. It is well established in the literature that the WSS influence the cardiovascular development and remodeling [4] and our results highlighted that both in R1 and R2 the combined action of the two jets gave rise to high values of the shear forces concentrated in the same areas of the left atrium (Fig. 11 and the values of $\overline{{\text {WSS}}_{\text {W}}}$ in Table 1). This could be relevant since the atrium is stressed by the regurgitant jets in the same areas during the heart remodeling, suggesting that in MVR due to a leaflet prolapse the left atrial size should be monitored by investigational tests in order to prevent the risk of an excessive dilation of the wall leading potentially to atrial fibrillation [79, 13, 71], the most common complication of the MVR, or in more rare cases to aneurysm formation [83, 87].

In the regurgitant cases we also noticed elevated values of WSS on the mitral valve leaflets, in correspondence of the free margin (Fig. 10 and the values of $\overline{{\text {WSS}}_{\text {MV}}}$ in Table 1). While the action of the WSS on the vascular tissue is quite understood, its effects on the valvular tissue are still under debate [8]. A shared conjecture is that the repeated action of the shear forces at every cardiac cycle could damage the endothelial valve tissue and trigger numerous active mechanisms resulting in potential valve degeneration and calcification [8].

## Final Remarks and Limitations

The main outcomes and contributions of the present study could be summarized as follows:

1. We showed how it is possible to build a framework to obtain virtual scenarios in the moving ventricle to analyze systolic hemodynamics, without the need of using a FSI model and by using instead dedicated dynamic images of the left ventricle and of the mitral valve; At the best of our knowledge, we described, for the first time, the presence of transition to turbulence in the atrium during systole in the regurgitant cases by means of a computational study;
2. We compared two possible regurgitant scenarios providing a quantitative comparison of different hemodynamic quantities, highlighting the ability of the system to compensate MVR and recover a physiological CO (albeit slightly smaller than the healthy case);
3. We quantified the chaotic velocity pattern in the atrium in the regurgitant cases highlighting the presence of two distinct jets; moreover, we assessed the WSS magnitude on the mitral valve and atrial wall, focusing on the increase of viscous forces due to regurgitation.

Some limitations characterized this study:

1. Starting from a ventricular geometry segmented from MRI, we filled the missing geometric data by considering a template for the left atrium and the aortic valve and a flow extension for the aorta. We notice however that such information are not in general visible from standard short-axis MRI acquisitions. For this reason, we are currently investigating the integration of short axis images with other MRI acquisition series that include also the left atrium and the aorta. Another geometric limitation consists in the smoothed ventricular endocardial reconstruction which did not include the papillary muscles, whose effect should be investigated in order to assess their influence in the quantities of interest of this study.
2. We expanded harmonically the ventricular displacement to the wall of the left atrium and aorta. With this choice we neglected the dilation of the left atrium and aorta during the ventricular systole. This point is currently under scrutiny, to understand how to include plausible dilation motions of aorta and left atrium, following e.g. the strategy proposed in Ref. [90].
3. We simulated only systole, including the isovolumic contraction for R1 and R2, since MVR is a pathology involving mostly this phase. Further investigations to extend our study to the diastolic phase will be mandatory in order to assess the effects of MVR during this phase and to remove the influence of the null initial condition more heartbeats should be performed. Notice that, we had nine systolic frames at our disposal, but in fact we worked only with six of them, and discarded three instants, since they were affected by a huge amount of noise that yielded a reconstruction that was incompatible with the other six, with a consequent irregularity of the displacement field and distortion of the mesh during its movement. Notice that these three frames were intermediate ones, thus we guaranteed to work with the patient specific SV.
4. We are aware that, in the clinical setting, the degree of MVR, for the same type of valvular disease, is extremely variable, depending upon several variable factors, such as heart rate, ventricular contractility, pre-load and after-load. Our current study was limited to the description of hemodynamics considering only one degree of MVR and two possible scenarios (increased heart rate and increased ventricular dimensions), maintaining all other parameters unchanged in all groups. The effects of different degrees of MVR on the hemodynamic quantities could be of clinical interest and investigated in future works, both conducting parametric studies accounting for a synthetic reduction/dilation of the mitral orifice, or analyzing several patients with different regurgitation severity.
5. In this work, we created three different virtual scenarios starting from a reference case. This is a well accepted strategy when one wants to compare the effect of a single change (in our case mitral regurgitation) on some outputs (see e.g. Ref. [35] for the case of mitral valve prolapse or Ref. [29] for different SAM degrees). The definition of these scenarios has the advantage of isolating specific pathological features of MVR from concurrent ones. The comparison between H and R1/R2 allowed us to assess the effects of the mitral regurgitation solely (together with the functional changes that this pathology implies, i.e. increased heart rate or dilation, according to the case) without introducing concurrent effects due to the different patients’ anatomies. Accordingly, we considered a single geometry, obtained from a single patient.

## Appendix: Mesh and Time Sensitivity Analysis

In this section, we reported the mesh and time sensitivity analysis performed on the healthy scenario. In particular, we considered the magnitude of WSS acting on the ventricular endocardium at the peak instant $t_{{\text {v}}1}$, see Fig. 6. We calculated the space average value ${\text {WSS}}_{\text {avg}}$ over all the ventricular endocardial points and we computed the relative difference $\Delta$ between this quantity obtained in two different meshes which differ from a number of elements of about 10%. In Table 2 we reported the mesh sensitivity analysis. After, we performed a time sensitivity analysis starting from $\Delta t = 0.25$ ms, by halving this value, and calculating the relative difference $\Delta$. in Table 3, we reported the time sensitivity analysis.

**Table 2:** Mesh sensitivity analysis performed on the meshes ${\text{H}}_{1}$ and ${\text{H}}_{2}$

Mesh	$\#$ Hexaedra	${\text {WSS}}_{\text {avg}}\ ({\text {Pa}})$	$\Delta \ (\%)$
${\text {H}}_{1}$	400636	0.2090	–
${\text {H}}_{2}$	438226	0.2050	1.9

**Table 3:** Time sensitivity analysis performed on mesh ${\text {H}}_{1}$

$\Delta t\ ({\text {ms}})$	${\text {WSS}}_{\text {avg}}\ ({\text {Pa}})$	$\Delta \ (\%)$
0.250	0.2090	–
0.125	0.2115	1.2

From both these sensitivity analyses we observed that the value $\Delta$ of the discrepancy on ${\text {WSS}}_{\text {avg}}$ is small in comparison to the refinement performed over space and time discretization parameters. Accordingly, the numerical simulations were performed with mesh ${\text{H}}_{1}$ and $\Delta t\ =\ 0.25$ ms.

## Footnotes

1 Notice that for the regurgitant cases R1 and R2 (see below) we also considered the isovolumic contraction when both the valves are closed, see “Inclusion of Left Atrium, Aortic Valve, and Aorta” section.

2 Provided by the Cine-MRI of the left ventricle, see “Acquisition of Cine-MRI Images” section.

3 with the symbol $\,\,\widehat{}\,\,$ we refer to the ED reference configuration.

4 When $\Gamma _{i}$ is a subscript, we omitted the temporal index $n+1$ for sake of clarity.

5 Notice that the displacement of the valves due to the ALE treatment of the *annuli* is included in the finite elements RIIS matrix and thus it should be not included in ${\mathbf {u}}_{\Gamma }.$
